# Protective effect of *Lactobacillus*-containing probiotics on intestinal mucosa of rats experiencing traumatic hemorrhagic shock

**DOI:** 10.1515/biol-2021-0112

**Published:** 2021-10-11

**Authors:** Lei Wang, Shu-li Liu, Zhi-peng Xu, Qi Song, Lei Li, Zhao-lei Qiu, Zhen-jie Wang

**Affiliations:** Department of Emergency, Anhui Provincial Hospital, Cheeloo College of Medicine, Shandong University, Jinan, Shandong, 250012, China; Department of Intensive Care Unit, The Second Affiliated Hospital of Shandong First Medical University, Tai’an, Shandong, 271000, China; Department of Emergency Surgery, The First Affiliated Hospital of Bengbu Medical College, Bengbu, Anhui, 233004, China; Department of Emergency Surgery, The First Affiliated Hospital of Bengbu Medical College, No. 287, Changhuai Road, Bengbu, Anhui, 233004, China

**Keywords:** compound *Lactobacillus*, trauma, mucosal integrity, NF-κB signaling pathway

## Abstract

This study was conducted to assess whether *Lactobacillus*-containing probiotics could protect intestinal mucosa in rats during traumatic hemorrhagic shock and to determine its underlying mechanisms. Healthy male Sprague–Dawley rats (300 ± 20 g) were randomly divided into four groups. During the study, reverse transcription polymerase chain reaction, western blotting, and hematoxylin and eosin methods were used. There was a significant increase in the expression of toll-like receptor 4 (TLR4) in the rats that experienced traumatic hemorrhagic shock, along with increased mRNA of tumor necrosis factor-alpha (TNF-α) and interleukin (IL)-6. Pretreatment with *Lactobacillus*-containing probiotics reduced TLR4 expression, decreased phosphorylation (Ser536) and acetylation (Lys310) of p65, and decreased TNF-α and IL-6 mRNA. The probiotics combined acetate Ringer’s group showed a less severe pathological manifestation compared to the other experimental groups. *Lactobacillus*-containing probiotics inhibited nuclear factor-kappa B signaling via the downregulation of TLR4, resulting in inflammatory homeostasis, which might be the mechanism whereby *Lactobacillus* protects the intestinal mucosa from damage caused by the traumatic hemorrhagic shock.

## 1 Introduction

According to the World Health Organization, 10% of deaths can be attributed to trauma [1], and globally trauma is the leading cause of death among people under the age of 40 years. Importantly, hemorrhagic shock is the leading cause of death in trauma patients [2]. Hemorrhagic shock leads to an uncontrolled inflammatory response in which many interleukins (IL), tumor necrosis factor (TNF), and other inflammatory mediators are released, eventually causing multiple organ failure (MOF). Intestinal dysfunction plays a pivotal role in the development of MOF since the integrity of the intestinal mucosal barrier prevents bacteria, antigenic agents, and toxins from entering the blood [3]. A study by Mesejo et al. suggested that increased intestinal permeability is one of the mechanisms implicated in MOF [3]. In addition, bacterial translocation that leads to systemic infection can promote the development of hemorrhagic shock.

*Lactobacillus*-containing probiotics are commonly used in current research due to their ability to optimize the intestinal microbiota composition, improve intestinal immune regulation, and suppress oxidative stress [4]. In recent years, the anti-inflammatory action mediated by *Lactobacillus*-containing probiotics has attracted tremendous attention [5]. These probiotics have been reported to have good therapeutic potential in treating diarrhea, inflammatory bowel disease, and acute pancreatitis [6,7,8], and the primary mechanism might be through the modulation of the nuclear factor-kappa B (NF-κB) signaling pathway mediated by toll-like receptor 4 (TLR4) [9]. TLR4 can recognize a wide variety of ligands to generate an inflammatory response and modulate immune homeostasis. Previous studies have demonstrated that the TLR4/myeloid differentiation factor-2 (MD2)/NF-κB pathway is abnormally activated when the intestinal barrier is damaged and that inhibiting the TLR4/MD2/NF-κB pathway can improve the barrier function [10]. In addition, the TLR4/NF-κB/mitogen-activated protein kinase signaling pathway was suggested to participate in the induction of ulcerative colitis, and that suppression of this pathway downregulated inflammatory cytokines, including IL-1β, IL-6, and TNF-α in colonic tissues [11]. However, the effect of *Lactobacillus*-containing probiotics on trauma-induced hemorrhagic shock remains unknown. Hemorrhagic shock due to trauma can directly injure the bowels and cause ischemia reperfusion injury of the intestine, leading to increased bacterial translocation and subsequent hyperinflammation [12]. This study was conducted to explore the protective effect of *Lactobacillus*-containing probiotics on the intestinal mucosa in rats experiencing traumatic hemorrhagic shock (THS) and to study the underlying mechanism of protection to reveal new avenues for the clinical treatment of THS.

## 2 Materials and methods

### 2.1 Equipment and reagents

Acetate Ringer’s (AR) solution was purchased from Hunan Kangyuan Pharmaceutical Co. Ltd (Hunan, China). TRIzol, UltraPure Agarose, Sybr qPCR mix, and the SuperScript reverse transcription (RT) kit were purchased from Invitrogen (California, United States). Primary antibody diluent and secondary antibody diluent, as well as p65 (Ser536) and p65 (Lys310) antibodies, were purchased from MDL Co. Ltd (Beijing, China). *Lactobacillus*-containing probiotics (Juke, Meitong Pharmaceutical Co. Ltd, Jiangsu, China) consisted of *Lactobacillus acidophilus*, *Streptococcus lactobacillus*, and more, with a viable count of >10^9^ colony-forming units (CFU)/g, with 0.33 g per capsule obtained from Greencross (Japan).

### 2.2 Animals

About 32 male specific pathogen-free Sprague–Dawley rats (300 ± 20 g) were purchased from Shanghai Jiesijie Laboratory Animal Co. Ltd (Shanghai, China). All the rats were raised under a temperature-controlled (22 ± 1°C) 12 h light–dark cycle with 55–45% humidity in a well-ventilated room.

The rats were randomly divided into four groups, with eight rats in each group. Rats in the control group were normal, untreated controls. Rats in the THS group experienced THS but did not receive fluid resuscitation. Rats in the THS + fluid replacement (FR) group experienced THS and were given FR using AR. Rats in the *Lactobacillus* group were pretreated with *Lactobacillus*-containing probiotics in drinking water for seven days prior to establishing THS and received a FR.


**Ethical approval:** The research related to animal use has complied with all relevant national regulations and institutional policies of the Bengbu Medical College for the care and use of animals.

### 2.3 Experimental procedures

#### 2.3.1 Experiment set-up

The rats were anesthetized by an intraperitoneal injection of 4% chloral hydrate (1 mL/100 g). The anesthetized rats were anchored to the operating table in a supine position. Skin from the bilateral inguinal region was prepared and disinfected with iodophor three times prior to towel placement. The bilateral femoral arteries and femoral veins were dissected and separated, respectively catheterized and fixed, and a small amount of 2.5% sodium citrate–glucose was injected to ensure the vessel was not blocked. The Medlab-U/2CS biological signal acquisition system (with zero setting and transducer calibration before use) was connected to the right femoral artery catheter to continuously monitor the mean arterial pressure (MAP). During all the procedures, normal saline (5 mL/kg/h) was injected via the femoral vein using a micropump to compensate the fluid loss from the surgical area and the respiratory tract.

#### 2.3.2 Establishment of hemorrhagic shock

The induction of hemorrhagic shock began 20 min after catheterization. At first, a sodium citrate solution (0.2 mL) was preloaded in a 2 mL syringe. Blood was discharged from the left femoral artery at a rate of 2 mL/3 min, and the MAP was maintained at 40–45 mmHg for 20 min. During the shock stage, the MAP was maintained at 40–45 mmHg via a small amount of bleeding or autologous blood transfusion for 60 min.

#### 2.3.3 Fluid resuscitation

The right femoral vein was used for fluid resuscitation after shock, and the left femoral vein was connected to a micropump. The rats in the THS + FR group and *Lactobacillus* group were resuscitated within 30 min using the AR solution. Resuscitation fluid was injected at a rate of 3:1 (resuscitation fluid to blood loss volume). No fluid resuscitation was administered in the THS group, and the rats were strictly observed for 4 h.

#### 2.3.4 Tissue collection

If the animals died within 4 h, tissues were collected immediately. The remaining rats were killed by cervical dislocation after 4 h of resuscitation, and the small intestine tissues were collected. The tissues were divided into three parts for quantitative PCR, western blotting, and histological examination.

### 2.4 Histological examination

Tissues were fixed in 4% paraformaldehyde solution. Hematoxylin and eosin (H&E) staining [13] was performed for pathological examination of the small intestinal tissue from the rats that experienced hemorrhagic shock and the appropriate control animals. Slides were observed under a light microscope. The pathological injuries in the intestinal tract were scored by a pathologist.

### 2.5 Quantitative RT-PCR

qRT-PCR was used to detect the mRNA expression of TNF-α, IL-6, and IL-10 in the small intestinal tissues harvested from the experimental rats. After obtaining small intestine tissue samples, total RNA was isolated and extracted by the Trizol method. RNA was reversely transcribed into the cDNA as per instructions on the Invitrogen kit, and then qPCR was done for amplification. glyceraldehyde phosphate dehydrogenase as the amplification primer: F: 5′-CCTCTATGCCAACACAGT-3′, R: 5′-AGCCACCAATCCACACAG-3′; TNF-α, F: 5′-GACTCTGACCCCCATTACTCT-3′, R: 5′-TGTTTCTGAGCATCGTAGTTGT-3′; IL-6, F: 5′-CACCCACAACAGACCAGTA-3′, R: 5′-GAAGCATCCATCATTTCTTT-3′; IL-10, F: 5′-GACAACATACTGCTGACAGATTC-3′, R: 5′-GCTGTATCCAGAGGGTCTTC-3′; miR-146a, F: 5′-GGGGGGTGAGAACTGAAT-3′, R: 5′-TCGTATCCAGTGCGTGTC-3′. At 95°C for 1 min pre-denaturation, 95°C for 15 s denaturation, 60°C for 30 s extension, cycling for 40 times; the dissolution curves were drawn (95°C, 1 min−95°C, 15 s−60°C, 30 s). After the reaction, the CT value was read and the mRNA expression was calculated.

### 2.6 Western blotting

Western blot was used to determine the protein expression of TLR4 and the phosphorylation (Ser536) and acetylation (Lys310) of the p65 subunit of NF-κB. About 100 mg of frozen small intestine tissue was taken, washed twice with phosphate buffered saline, cut into pieces, and then 1 mL of radio immunoprecipitation assay lysate was added and ground on ice. After centrifugation at 12,000 rpm for 10 min in a 4°C centrifuge, the supernatant was taken for quantitative analysis of BCA protein. Sodium dodecyl sulphate–polyacrylamide gel electrophoresis was performed with 10% separation gel and 4% concentrated gel, and then the gel was transferred to the PVDF membrane by the wet membrane transfer method. The 5% skim milk powder sealant was placed on a shaker for blocking for 2 h, and the corresponding primary antibody was added. After incubation overnight at 4°C, the membrane was washed by tris buffered saline tween (TBST) for three times. The secondary antibody was added and incubated at room temperature for 1 h, and then the membrane was washed by TBST for three times. Finally, the film was exposed, developed, and fixed in a darkroom by enhanced chemiluminescence and analyzed with Fluorchem gray analysis software. The grayscale ratio of the target protein to β-actin is the relative expression level of the protein.

### 2.7 Statistical analysis

All data were analyzed using SPSS v22.0, and the results presented as mean ± standard deviation. One-way analysis of variance was used to compare the data between groups. Fisher’s least significant difference test was used for pairwise analysis. A *P* value of <0.05 was considered statistically significant.

## 3 Results

### 3.1 mRNA expression of inflammatory factors in the tissues from the small intestine of rats that experienced hemorrhagic shock

**Figure 1:**
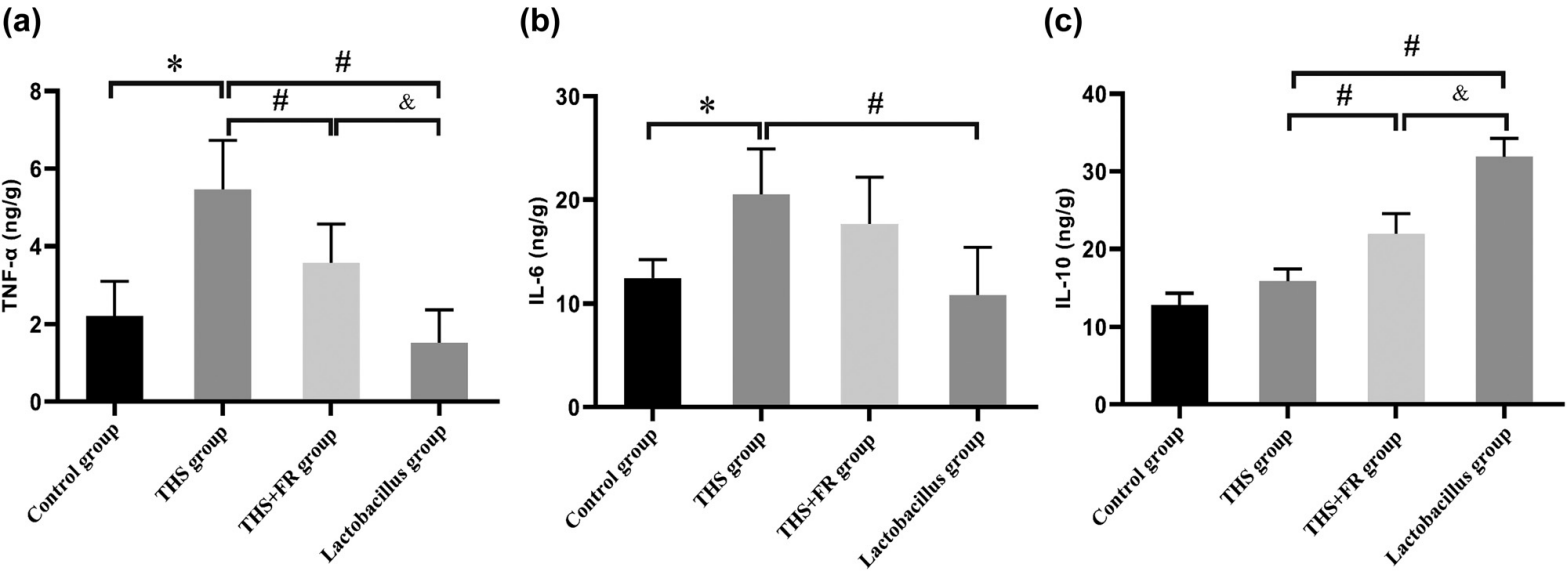
The mRNA expression of TNF-α (a), IL-6 (b), and IL-10 (c) in different groups. Differences in the levels of mRNAs were detected by qPCR; *n* = 8; *P < 0.05, compared with the control group; ^#^ *P* < 0.05, compared with the THS group; and ^&^ *P* < 0.05, compared with the THS + FR group.

The PCR analyses revealed that compared to the control group, the mRNA expression of TNF-α and IL-6 had significantly increased. Nevertheless, when compared with the THS and THS + FR groups, the mRNA of TNF-α had significantly decreased in the *Lactobacillus* group (*P* < 0.01 respectively; Figure 1a). Similarly, IL-6 mRNA was also significantly reduced in the *Lactobacillus* group compared to the THS + FR groups (*P* < 0.05 respectively; Figure 1b). On the other hand, the expression of anti-inflammatory factor IL-10 in the *Lactobacillus* group was significantly higher than that in the THS group and the THS + FR group (*P* < 0.01 respectively; Figure 1c).

### 3.2 TLR4 expression in the small intestine

**Figure 2:**
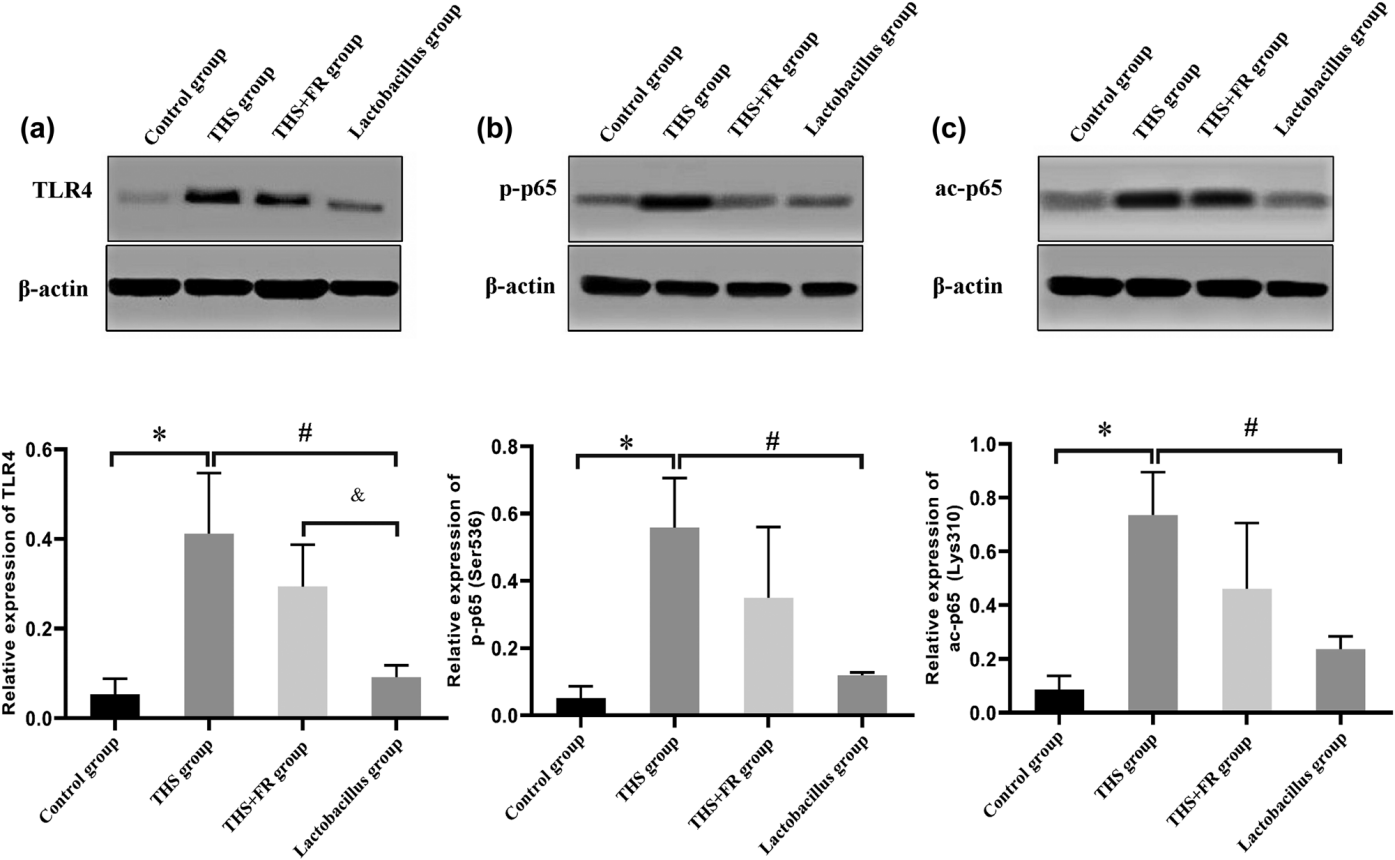
Protein expression of TLR4 (a), p65 phosphorylation (Ser536) (b), and acetylation (Lys310) (c) in tissue from the small intestinal. Differences in the levels of proteins were detected by western blot; *n* = 8; *P < 0.05, compared with the control group; ^#^ *P* < 0.05, compared with the THS group; and ^&^ *P* < 0.05, compared with the THS + FR group.

The western blot results showed that the TLR4 protein expression had significantly increased in the THS group compared to the control group (*P* < 0.01). In the *Lactobacillus* group, TLR4 expression had decreased compared to the THS group and the THS + FR group (*P* < 0.01) (Figure 2a).

### 3.3 Phosphorylation (Ser536) and acetylation (Lys310) of NF-κB p65 in the small intestine

Compared to the THS group, the phosphorylated p65 (Ser536) and acetylated p65 (Lys310) levels in the *Lactobacillus* group had significantly decreased (*P* < 0.05; Figure 2b and c).

### 3.4 Histopathology of the small intestine

**Figure 3:**
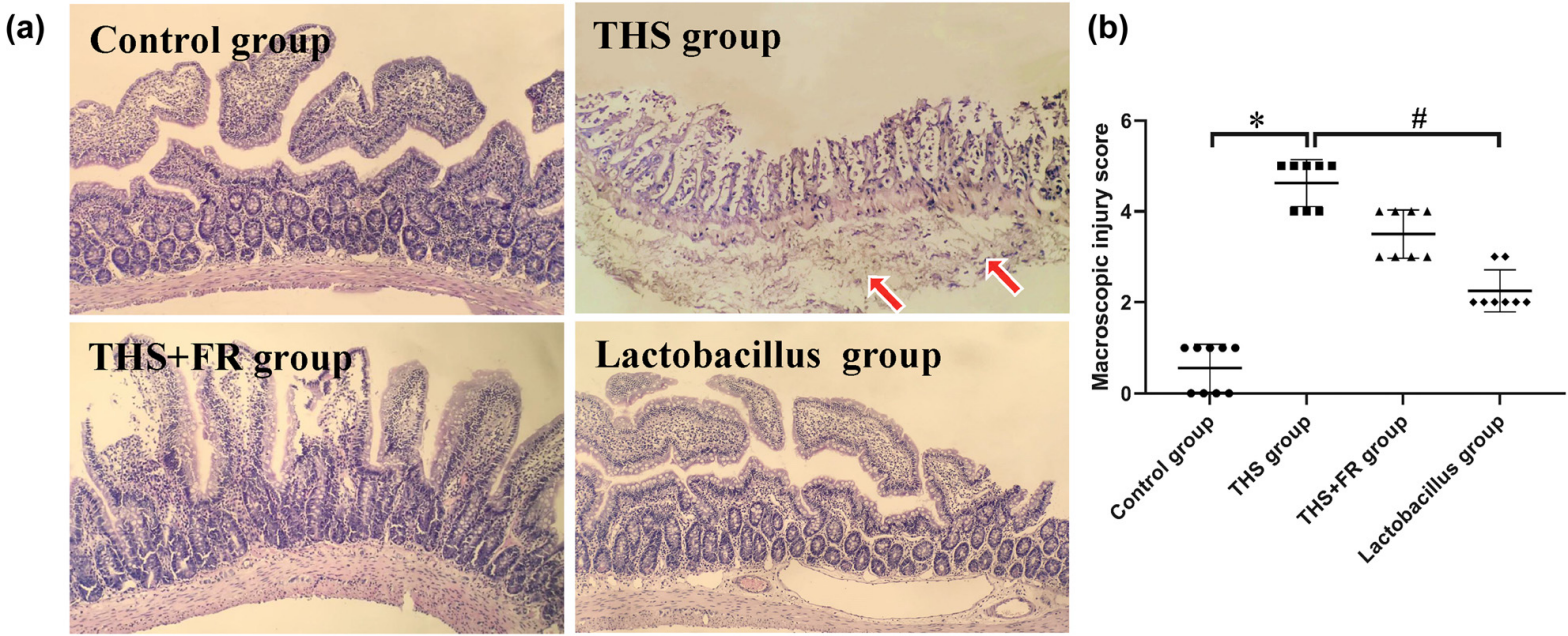
Histopathology of the small intestine. (a) The histopathology of the small intestine observed under light microscopy (H&E; ×10), red arrow indicates intestinal mucosal damage; (b) macroscopic injury score; *n* = 8; *P < 0.05, compared with the control group; ^#^ *P* < 0.05, compared with the THS group.

The control group displayed intact intestinal mucosa, well-arranged glands, and normal intercellular space. In the THS group, the subepithelial space of the villi was enlarged and accompanied by a separation of the upper cortex and lamina propria. The capillaries of the villi were congested, while a part of the villus tip was damaged. The lamina propria was bleeding, ulcerated, and infiltrated with inflammatory cells. Compared to the THS group, the villi in the THS + FR group showed expansion of the subepithelial space with moderate separation of the upper cortex and lamina propria, and some tips of the villi were slightly damaged. Compared to the THS group and the THS + FR group, the intestinal structure of each layer of the *Lactobacillus* group was relatively complete, and the villi were arranged in an orderly manner. The turbidity and swelling of the small intestinal cells were significantly reduced, while no obvious cell necrosis was observed (Figure 3a). In addition, the scores of the pathological injury of the intestinal tract in the THS group were higher than in the control group and that of the *Lactobacillus* group were significantly lower than in the THS group (Figure 3b).

## 4 Discussion

THS leads to hypovolemia and hypoperfusion of internal organs. For its own protection, the body prioritizes sending blood to the brain, heart, and kidneys to maintain normal function. This greatly reduces blood supply to the gastrointestinal tract, the first organ system affected by hemorrhagic shock [14]. Ischemia of the mucosa can damage the intestinal mucosal barrier and cause intestinal epithelial dysfunction. Subsequently, bacteria of the intestinal microbiota can penetrate the barrier and induce systemic inflammatory response syndrome, which can further develop into MOF [15]. Importantly, TLRs play a pivotal role in mediating the immune response. TLR4 was the first TLR discovered and is the most well-studied family member [16]. Like many receptors, TLRs link extracellular immune stimulation to an intracellular immune response, and with TLR4 specifically, ligand binding can lead to the activation of the NF-κB signaling pathway. Under normal circumstances, TLR4 is rarely expressed in intestinal mucosa. However, dysregulation of the microbiota can disrupt host intestinal mucosal immune tolerance and lead to increased expression of TLR4 on intestinal epithelial cells. Subsequently, excessive expression of TLR4 caused over-activation of NF-κB and increased release of inflammatory mediators such as TNF-α, IL-6, and IL-8, which can cause tissue injury [17] and even sepsis [18]. NF-κB is demonstrated to be a key regulatory gene responsible for intestinal damage secondary to hemorrhagic shock, and inhibition of its activation alleviates hemorrhagic shock and organ dysfunction [19,20]. Recently, studies have found that TLR4 overexpression also occurs during hemorrhagic shock, which may be related to the destruction of the intestinal mucosal barrier caused by the redistribution of blood flow and the ability of intestinal bacteria to penetrate the barrier following this damage [21].

Colonization of the intestinal tract with lactic acid bacteria improves microecological balance, assists the host in metabolizing nutrients, and regulates the host immune system [8]. Receptors such as TLRs play a vital role in host recognition of extracellular signals. Lee et al. suggested that lactic acid bacteria can reduce TLR4 expression while mitigating an intestinal inflammatory response [9]. Studies have also demonstrated that lactic acid bacteria can attenuate intestinal inflammation, inhibit the production of inflammatory cytokines, and downregulate the NF-κB signaling pathway [9,10]. Monocytes and macrophages had also been shown to be stimulated by lactic acid bacteria to maintain immune homeostasis [22,23]. In this study, rats pre-treated with *Lactobacillus*-containing probiotics showed downregulated TLR4 expression compared to rats that did not receive the probiotics, which suggests that the *Lactobacillus*-containing probiotics inhibited TLR4 expression, thereby reducing the over-activation of the NF-κB signaling pathway to relieve intestinal inflammation.

NF-κB is the ultimate effector of TLR signaling and is usually present as an inactive complex consisting of the p50 and p65 subunits and the inhibitory protein IκBα [24]. We found that the phosphorylation and acetylation of p65 increased during hemorrhagic shock, but pretreatment with *Lactobacillus*-containing probiotics could reduce this increase. Our results suggest that *Lactobacillus*-containing probiotics can downregulate hemorrhagic shock-induced p65 phosphorylation in the intestine, thereby inhibiting NF-κB signaling.

TNF-α and IL-6 are important mediators of the immune response and chronic inflammation, and the gene promoter regions of both cytokines contain the NF-κB binding site. Thus, inhibiting NF-κB activation could downregulate the production of TNF-α and IL-6 as well as other inflammatory factors, which ultimately would result in less inflammation [25]. IL-10 is an anti-inflammatory cytokine that inhibits the inflammatory response and can improve disease prognosis [26]. Notably, the balance between inflammatory and anti-inflammatory responses determines disease progression. Recent studies have shown that *Lactobacillus*-containing probiotics can promote the release of IL-10 through the stimulation of multiple immune cells, such as dendritic cells, monocytes, and regulatory T cells [27]. Researchers observed that probiotics can mitigate inflammatory bowel disease through the regulation of IL-10 [28]. In this study, the expression of TNF-α and IL-6 mRNA was significantly increased in the rats experiencing hemorrhagic shock, indicating the severity of the host’s inflammatory response secondary to hemorrhagic shock. However, pre-treating the rats with *Lactobacillus*-containing probiotics was able to decrease the levels of TNF-α and IL-6 mRNA. Consistent with these finding, phosphorylated (Ser536) and acetylated (Lys310) p65 were also downregulated by *Lactobacillus*-containing probiotics. At the same time, IL-10 was up-regulated, which balanced the inflammatory response and therefore reduced likely intestinal injury.

## 5 Conclusion

We concluded that *Lactobacillus*-containing probiotics can inhibit the activation of the NF-κB signaling pathway by downregulating the expression of TLR4, and can protect the intestinal mucosa of rats suffering hemorrhagic shock by reducing the proinflammatory cytokines IL-6 and TNF-α while increasing the levels of IL-10. Nonetheless, hemorrhagic shock is much more complex in human patients with multiple pathological factors involved. Furthermore, the preparation of *Lactobacillus* and the timing of its clinical application are still being debated. So, further research is required to optimize the therapeutic protocol to accelerate the clinic translation process.
